# Lessons learned from implementing a large-scale contact investigation and TB preventive treatment project in India

**DOI:** 10.5588/ijtldopen.26.0168

**Published:** 2026-08-11

**Authors:** B. Kalottee, A. Nuken, A. Kalra, S.S. Chadha, S. Mannan, M. Singh, H. Solanki, R. Taralekar, M. Yassin, N. Restrepo, R. Rao, S.K. Mattoo, V. Dhawan, S. Rajan

**Affiliations:** 1 International Union Against Tuberculosis and Lung Disease, South-East Asia Office, New Delhi, India;; 2 FIND India, New Delhi, India;; 3 William J Clinton Foundation, New Delhi, India;; 4 World Health Organization (WHO), Country Office for India, New Delhi, India;; 5 Global Fund to Fight AIDS, Tuberculosis and Malaria, Geneva, Switzerland;; 6 Central TB Division, Directorate General of Health Services, Ministry of Health & Family Welfare; New Delhi, India.

**Keywords:** tuberculosis, household contacts, TPT adherence, community health workers, implementation challenges

## Abstract

**BACKGROUND:**

TB preventive treatment (TPT) is central to reducing TB incidence among high-risk populations. This paper presents learnings from a large-scale TPT implementation under a Global Fund-supported project in India.

**METHODS:**

The project targeted household contacts (HHCs) of people with pulmonary TB (PTB) across 208 districts in 23 states. Data on symptom-based screening, TPT initiation, adherence, and completion were analysed to inform scale-up strategies.

**RESULTS:**

Of 3,097,831 HHCs identified from 851,974 people with PTB, 91% were symptom-screened, and among those screened 28% underwent chest X-ray and 54% initiated TPT, with an 84% completion rate. Integration with active case finding, portable digital chest X-rays, and short-course regimens (3HP) improved coverage. Decentralised service delivery through Health and Wellness Centres and engagement of community health workers enhanced treatment access and follow-up. Teleconsultations, digital tracking, and incentive mechanisms supported adherence. Challenges included early discontinuation due to adverse drug reactions, regional diagnostic disparities, and limited private sector engagement.

**CONCLUSION:**

Large-scale TPT delivery is feasible in high-burden settings when integrated with routine health system platforms and community-based approaches. Key operational insights included chest X-ray screening, and scale-up of shorter regimens, supporting India’s TB elimination goals.

TB remains one of the leading causes of morbidity and mortality in India, which accounts for nearly one fourth of the global TB burden.^1^ Despite substantial progress made under the National TB Elimination Programme (NTEP), the risk of transmission particularly within households of patients with pulmonary TB (PTB) continues to pose a significant public health challenge.^2^ The WHO recommends scale-up TB preventive treatment (TPT) to protect household contacts (HHCs), people living with HIV (PLHIV), and other vulnerable populations.^3^ Contact investigation (CI) and TPT are critical strategies to interrupt transmission and prevent progression from TB infection (TBI) to TB disease as endorsed by global and national guidelines.^3–6^ However, despite increasing policy support and investments, TPT implementation at scale remains limited. Programmatic bottlenecks persist across the TPT cascade, from diagnosis to treatment completion owing to variations in diagnostic algorithms, logistical constraints, limited awareness among health care providers and communities, and broader health system gaps including workforce shortages and inconsistent supply chains.^3,7,8^ Under the Global Fund’s sixth grant cycle (GC6: 2021–2024), strategic, financial, and operational support, India accelerated the TPT uptake through the rollout of shorter regimens (3HP [3 months of weekly isoniazid and rifapentine]) targeting HHCs and PLHIV.^9^

Despite evolving global and national policies on CI and TPT, operational evidence on their large-scale implementation in routine, resource-limited settings remains limited. Most studies emphasise clinical efficacy or small-scale pilots, offering little insight into practical challenges or adaptive strategies for TPT scale-up in diverse contexts like India.^10^ In India, persistent barriers such as low community acceptance, logistical constraints, workforce shortages, and erratic drug supply hinder effective TPT rollout. Gaps in private provider engagement, ambiguity around HHC eligibility, stigma, and limited diagnostic capacity further impede uptake and coverage.^8,11–13^ These challenges underscore the need for focused implementation to address operational gaps and strengthen the scale-up of TPT and CI strategies.

We present key lessons from implementing CI and TPT under programmatic conditions, highlighting innovative practices, enablers, and implementation challenges in high-burden settings.

## METHODS

This programme implementation analysis draws on routine programme data, programme reports, and stakeholder consultations to document operational lessons from implementing CI and TPT under the Global Fund Round 6 (GC6) grant (2021–2024).

### Study Setting

**Figure 1.**
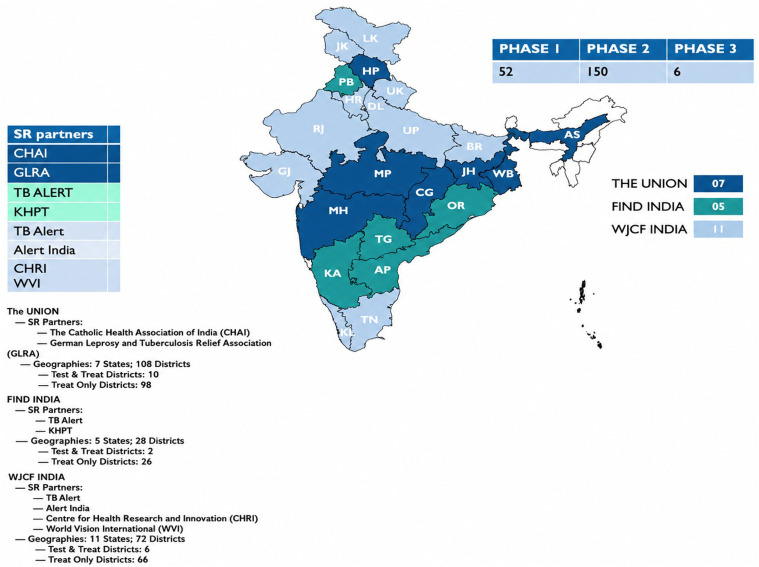
Geographic coverage under TB preventive treatment project.

**Figure 2.**
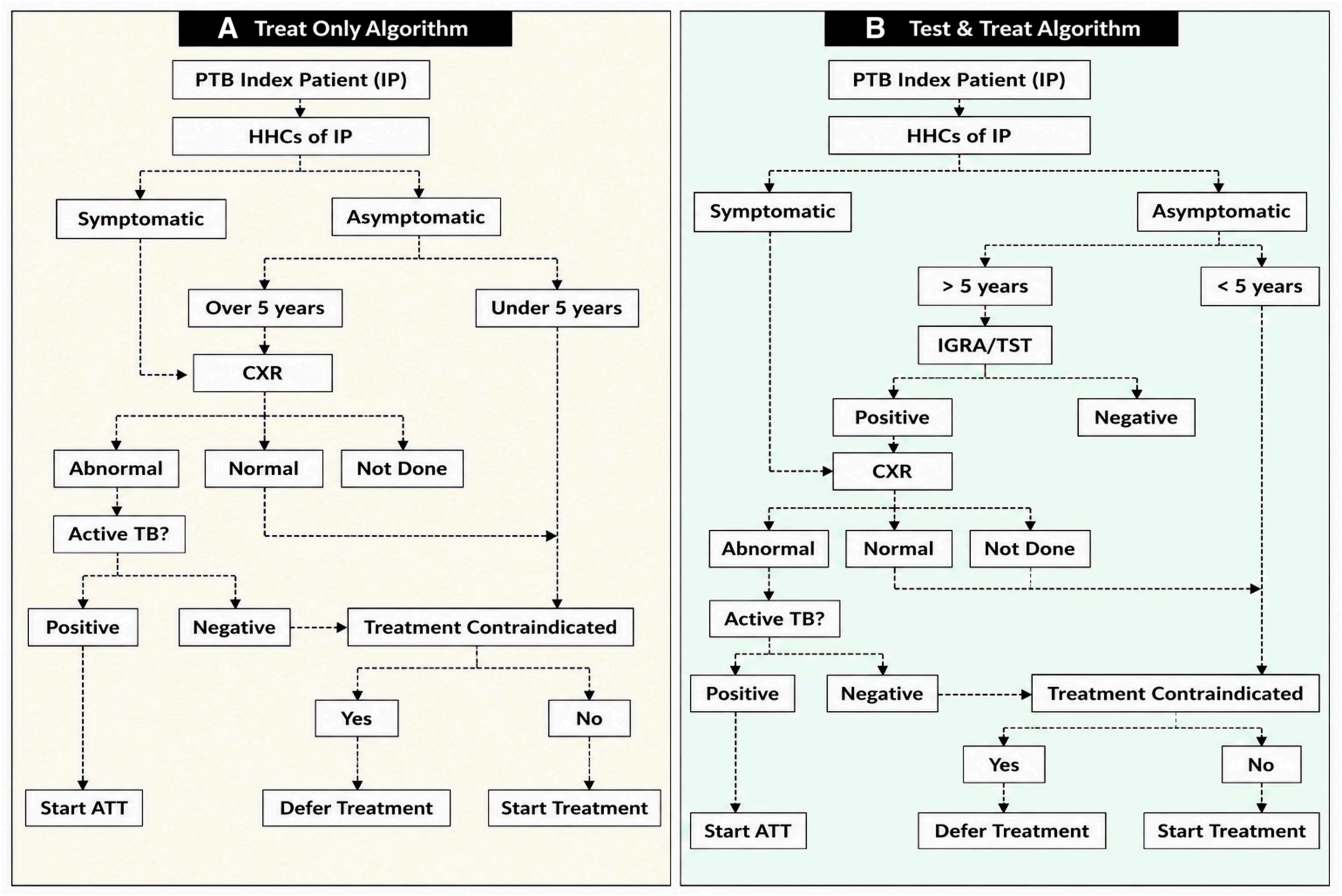
Algorithm for TB screening and providing TB preventive treatment (TPT) in India. ATT = anti-tuberculosis treatment; CXR = chest X-ray; HHC = household contact; PTB = pulmonary TB.

This project was implemented across 208 districts in 23 states of India, covering an estimated 400 million people (∼28% of the population). It was led by three principal recipients, International Union Against Tuberculosis and Lung Disease (The Union), Foundation for Innovative New Diagnostics (FIND), and William J. Clinton Foundation (WJCF), and six sub-recipients (local implementing partners) including CHAI, GLRA, TB ALERT, KHPT, ALERT India, CHRI, and WVI (Figure 1). The interventions were executed under Project Axshya Plus (The Union) and JEET 2.0 (FIND, WJCF), targeting HHCs of drug-sensitive PTB patients notified in Ni-kshay (the electronic database) identified through passive, intensified, or active case finding (ACF). Across the 208 districts included in the study, implementation approaches varied: 18 districts adopted a ‘Test and Treat’ strategy, while the remaining districts implemented a ‘Treat Only’ model, as illustrated in Figure 1. TPT implementation followed the NTEP algorithm with adaptations. Two delivery models were used (Figure 2): 1) In 18 districts, a ‘Test and Treat’ model included symptom screening (persistent cough [>2 weeks], fever, weight loss, and night sweats), chest X-ray (CXR), and interferon-gamma release assay (IGRA)/tuberculin skin test (TST) testing. Symptomatic individuals underwent CXR and diagnostic testing for TB disease (sputum/cartridge-based nucleic acid amplification test), while asymptomatic HHCs aged >5 years were tested for TBI (IGRA/TST).Those testing positive for TBI underwent further clinical assessment and CXR before initiating TPT (3HP, or 6H [6-month daily isoniazid]) as per PMTPT guidelines.^6^ 2) In 190 districts, a ‘Treat Only’ model offered TPT to eligible HHCs based on symptom screening and normal CXR findings and assessment for contraindications, without TBI testing.

### Data collection, variables, and source

Data were drawn from routine programme data, programme reports, and stakeholder consultations to analyse programme planning and implementation, including identification of implementation barriers, enablers, and lessons learned. Triangulation enhanced the validity and contextual interpretation of findings.

### Analysis approach

Descriptive analysis of routine programme data summarised key indicators on CI, diagnostic testing, and TPT initiation and completion. Technical and operational lessons were synthesised from programme reports and stakeholder consultations to identify implementation barriers, enablers, and adaptive strategies.

### Ethical statement

The study used anonymised routine programme data and stakeholder consultations with verbal consent. In line with the Indian Council of Medical Research (2017) guidelines, no formal ethical clearance was required. All procedures adhered to ethical standards, ensuring voluntary participation and confidentiality.

## RESULTS

A total of 851,974 people with PTB were visited across 208 districts, leading to identification of 3,097,831 HHCs for screening. Of these, 2,826,891 HHCs (91%) underwent symptom-based screening. CXR was performed for 781,180 HHCs to identify subclinical and radiological abnormalities consistent with TB and to rule out TB disease.

In the 18 Test and Treat districts, 75,649 HHCs were tested for TBI using IGRA, of whom 20,528 individuals (27%) tested positive. Overall, 1,539,497 HHCs (54% of those screened) were initiated on TPT according to national guidelines and programmatic eligibility.

Regimen-wise treatment outcome analysis was conducted among 993,464 HHCs with complete treatment outcome data across implementing partners and regimens. The overall treatment completion rate was 84%. Completion was high for short-course regimens, at 92% for both 3HP and 3HR; however, 3HR data were available only from a single implementing partner (The Union). The 6H regimen, which accounted for the majority of initiations, had a completion rate of 83%.

Across six JEET 2.0 pilot districts, IGRA positivity was comparable among HHCs of bacteriologically confirmed PTB (35.7%) and clinically diagnosed PTB (35.6%) following exclusion of active TB prior to TPT initiation. In a pre-specified Maharashtra sub-analysis (Axshya Plus districts), conducted due to complete disaggregated data availability, IGRA positivity was higher among contacts of bacteriologically confirmed PTB (25.6%) than clinically diagnosed PTB (19.5%) (*P* < 0.005) (Table 1). Marked geographical variation was observed in IGRA positivity, ranging from approximately 25% in southern districts to up to 50% in hilly regions, with additional with additional intra- and inter-district variation.

**Table 1.** Summary of key technical learnings and implications for national TB policies.

Technical finding	Key observations/learnings	Implications for national policies
1. IGRA positivity in HHCs of BCPTB and CCPTB	Similar IGRA positivity among HHCs in six JEET 2.0 pilot districts (35.7% in BCPTB, 35.6% in CCPTB), where TBI was ruled out through IGRA testing before initiating TPT. However in Maharashtra, IGRA positivity was found significantly higher among HHCs of BCPTB (25.6% in BCPTB, 19.6% in CCPTB). The comparable positivity across both groups indicates that IGRA positivity is not exclusive to bacteriologically confirmed TB cases.	Suggests revisiting national guidelines that prioritise household contacts of BCPTB cases for TB preventive services.
2. TPT screening and active case finding (ACF) synergy	Combined ACF and TPT screening led to identification of 3,850 new TB cases through ACF, with a 5% positivity rate among chest symptomatic individuals. 65% of TB diagnoses came from asymptomatic individuals, helping to rule out active TB before TPT initiation. Handheld X-rays used for CXR at the doorstep.	Supports the integration of ACF with TPT services to maximise case detection and streamline services.
		Promote doorstep diagnostic tools for early TB detection.
3. Chest X-ray integration	CXR improved TB detection and built trust for TPT uptake; 50% suggestive cases, 2% confirmed TB.	Institutionalise CXR screening in TPT algorithms; strengthen portable CXR access in peripheral and rural areas.
4. TBI testing: quality and cost-effectiveness	Significant variation in positivity from IGRA (45%–50%), dropping to TST (10%) in Himachal Pradesh when the State transitioned to TST for TBI testing, following withdrawal of IGRA services through Grant support. IGRA was costlier ($29/test), and TST’s accuracy varied due to lack of skilled staff.	Need to assess cost-effectiveness of IGRA versus TST, and consider introducing new WHO-approved tests like Cy-Tb for scalability.
5. Geographical variation in TB infection	IGRA positivity rates varied from 50% in hilly regions to 25% in southern India, with variations within states and districts.	Highlights need for context-specific diagnostic strategies when scaling TPT services in diverse geographies.
6. Engagement of general health system	Health and Wellness Centres (HWCs) played a major role in TPT initiation and completion, with ASHAs and community health workers improving adherence. Incentive schemes boosted treatment adherence.	Reinforces the importance of strengthening general health systems, decentralising TB services to HWCs, and incentivising workers.
7. Private sector engagement in TB prevention	Private sector uptake of TPT was low (17%). Despite efforts like continuing medical education, engagement remained a challenge, contrasting with higher uptake reported by other entities (JEET 2.0).	Calls for a distinct strategy to engage the private sector, potentially with provider-focused communication and incentives.
8. Long-term follow-up post-TPT completion	Long-term follow-up post TPT completion found a low rate of active TB diagnosis (<1%) after TPT completion, supporting the protective efficacy of TPT.	Emphasises the need for systematic long-term follow-up after TPT completion to assess effectiveness and prevent TB recurrence.
9. Care cascade	Every step from screening to completion is critical for achieving desired outcomes.	Develop digital dashboards and early-warning systems to monitor drop-offs and support real-time decision-making.
10. Counselling strategies	Counselling within the first month of index TB treatment was most effective; use of tailored tools (e.g., flipcharts, job aids, and videos) helped engagement.	Institutionalise structured counselling protocols and materials; prioritise early engagement of HHCs.
11. Shorter regimens	3HP had higher treatment completion (91%) and acceptability than 6H (86%).	Expand 3HP availability nationally; consider patient preferences and context-specific implementation strategies.
12. Adverse drug reaction (ADR) management	ADRs (8%–17.5%) particularly in the first month of the treatment were a major cause of treatment discontinuation; proactive management improved adherence.	Develop ADR management SOPs; integrate teleconsultation for ADR management and follow-up through telemedicine platforms to enable timely interventions specially in hard-to-reach and underserved areas.

Bacteriologically confirmed pulmonary tuberculosis (BCPTB); clinically confirmed pulmonary tuberculosis (CCPTB); IGRA = interferon-gamma release assay; HHCs = household contacts; TBI = TB infection; TPT = TB preventive treatment; CXR = chest X-ray.

Integration of CI with ACF improved TB detection, identifying 3,850 bacteriologically confirmed TB cases among HHCs. Among chest symptomatic individuals tested, the positivity rate was 5%. Notably 65% of TB diagnoses were among asymptomatic contacts, which supported exclusion of active TB before initiation of TPT. CXR improved diagnostic confidence, with 50% showing suggestive findings and 2% confirmed TB. Use of handheld digital X-ray at household level enabled identification of both presumptive and asymptomatic TB cases.

**Figure 3.**
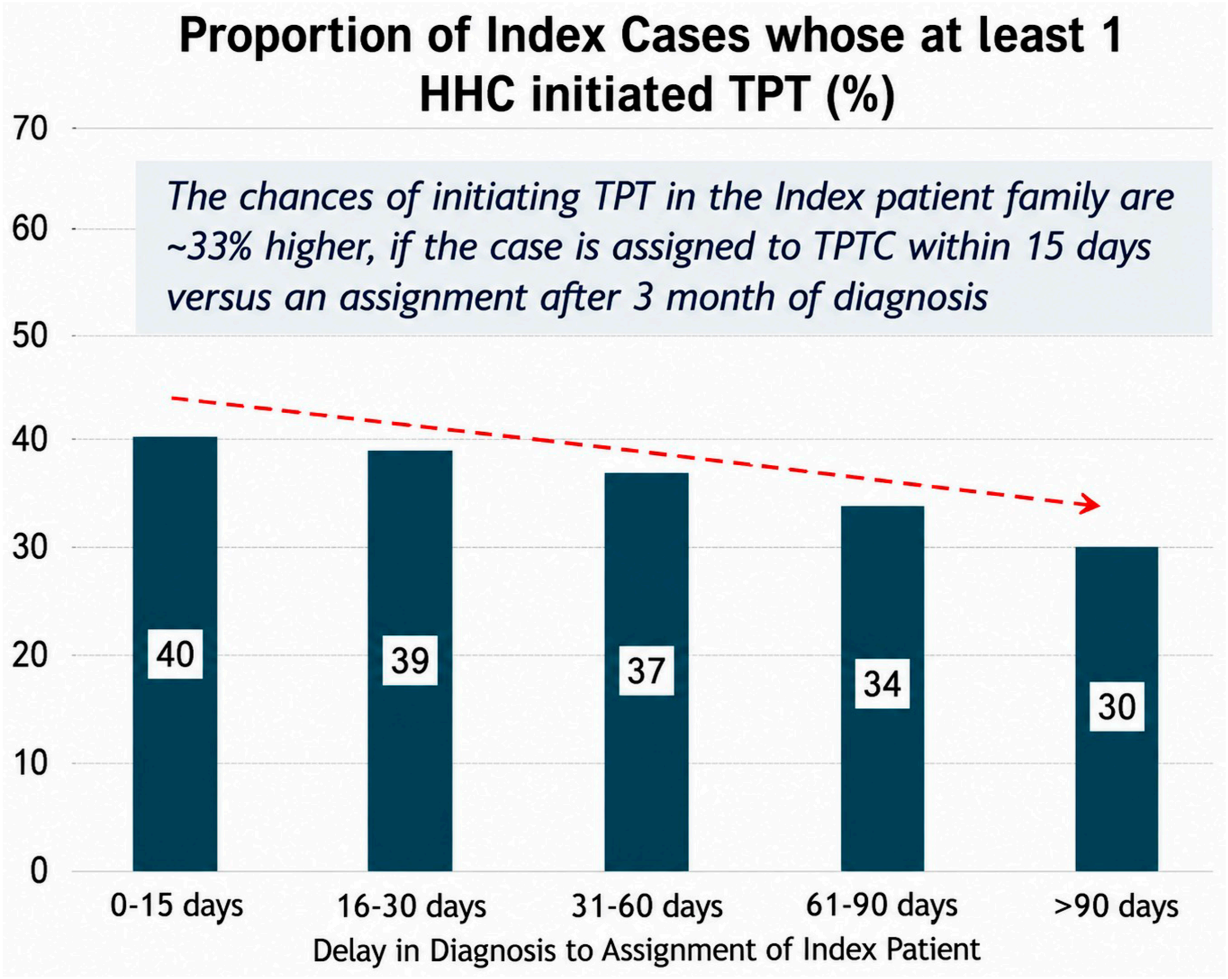
Delay in diagnosis to assignment of index patient to TB preventive treatment coordinators (TPTC). HHC = household contact; TPT = TB preventive treatment.

Timeliness of assignment to TPT coordinators (TPTCs) was a key determinant of initiation. HHCs were approximately 33% more likely to initiate TPT when index cases were assigned to TPTCs within 15 days compared to delays exceeding 3 months (Figure 3). Strengthening of digital case allocation, field deployment, and contact tracing increased household coverage from 28% to 98% over the implementation period (Table 2). Decentralised service delivery, including doorstep screening, testing, and drug distribution, reduced access barriers and improved programme reach. Households served through decentralised or near-home diagnostic models experienced minimal pre-treatment loss to follow-up, whereas reliance on centralised facilities was associated with attrition of up to 40%. Health and Wellness Centres (HWCs), initially underutilised, became key delivery points for TPT following targeted capacity building and coordination by District TB Officers and Chief Medical Officers.

**Table 2.** Operational challenges, strategies, and outcomes in scaling up TB preventive treatment (TPT).

Theme	Challenge identified	Operational strategy/innovation	Impact/outcome
1. Post-diagnosis delays in contact tracing and TPT Coordinator (TPTC) linkage lead to missed TB prevention opportunities	Delay between diagnosis of the index TB patient and case assignment to the TPTC is inversely related to TPT initiation rates among household contacts (Figure 3).	- Define and enforce time-bound protocols for each step in the TPT cascade, especially for case assignment.	Districts that ensured TPTC assignment within 15 days of diagnosis reported ∼33% higher TPT initiation rates than those with delayed assignment (>3 months). Improved cascade efficiency with higher contact screening and treatment initiation.
		- Strengthen digital platforms like Ni-kshay for real-time case allocation and automatic alerts to TPTCs.	
		- Integrate timeliness indicators into routine monitoring.	Empowered community-level actors to close gaps in TPT delivery.
		- Decentralise and streamline processes by empowering frontline workers for timely follow-up.	
2. Reaching the unreached	Incorrect contact details, difficult geographies, refusal	- Deploying LTBI-C to TB units	Improved household reach and reduced time delays. The HHC visits improved from 28% to 98% within a year’s time after revising the strategy.
		- Night stay for LTBI-C in remote areas	
		- Team Lead coordinated directly at block level to update contact details of TB patient	
		- Updated Ni-kshay system for future references	
3. Addressing refusal (HHCs, HCWs, private providers)	Refusal due to lack of symptoms, fear, stigma, workload	- Developed tailored communication strategy	Screening improved to 87%; increased willingness to start TPT
		- Used checklist for counselling	
		- Trained staff in soft skills	
		- Involved ASHAs and TB survivors for counselling	
4. Improving TPT uptake	- MO-only TPT initiation, poor drug access, distant health facilities	- LTBI-C posted at TB Units to support HHCs to initiate TPT	Uptake improved from 17%; improved access, timely initiation
	- Drug stockouts	- Door step delivery of testing (IGRA and chest X-ray)	
		- Drug decentralisation to peripheral facilities	
		- Engaged CHOs and HWCs	
		- State-driven family call-in strategy	
5. Ensuring drug availability	TPT demand exceeded NTP supply, Drug interruptions leading to interruptions in TPT initiation, impact HHCs on TPT	- Drug forecasting tool developed	Prevented loss to follow-up, improved continuity of care
		- Local procurement supported by providing technical assistance	
		- Daily monitoring of drug availability	
6. Enhancing TPT outcome and adherence	Early dropouts (within first month of TPT initiation) due to adverse event (AEs), unmanaged AEs, lack of monitoring, entire family discontinue TPT if 1 member experience AE.	- Increased follow-ups (F/U) (physical and telephonic) – 7F/U (three physical and four telephonic) for 6H regimen	- Loss to follow-up reduced from 19% to 4%
		12 F/U (three physical and nine telephonic) for 3HP regimen	- Higher treatment completion
		- Prioritise early follow-up by ensuring at least two in-person monitoring visits during the first month of TPT to strengthen adherence	- Effective community and system engagement strategies for adherence
		- Engagement of CHOs, NTEP staff, community workers – ASHAs, Mitanins, Sahiyas	
		- Incentives to ASHAs (INR 250) and HHC on completion of TPT	
		- Tools: TPT cards (similar in design to routine immunisation cards), zip-lock pouches to store TPT drugs and count the number of doses taken, SMS reminders	
		- A four-tier retrieval model in Chhattisgarh – escalating counselling from the project team to ASHAs, then CHOs/NTEP staff, and finally the DTO – proved effective in re-engaging HHCs who discontinued TPT without medical advice, significantly reducing loss to follow-up	
7. Improving service delivery models	Limited doorstep diagnostic availability	- Doorstep delivery of IGRA and CXR screening (using handheld X-ray)	Increased TPT acceptance when preceded by diagnostic confirmation
8. Integrated TPT screening and ACF	Missed opportunities when TPT screening and ACF done separately	- Bundled TPT screening with ACF in households	Efficient contact screening and better yield of services

ACF = active case finding; CHO = community health officer; DTO = district tuberculosis officer; LTBI = latent tuberculosis infection; LTBI-C = latent tuberculosis infection coordinator; IGRA = interferon-gamma release assay; HHC = household contact; TPT = TB preventive treatment; CXR = chest X-ray; NTEP = National TB Elimination Programme.

Counselling delivered within the first month of index case treatment using structured tools, such as flipcharts, videos, and job aids, improved uptake. Effective counselling addressing timing (WHEN), target audience (WHO), and delivery approach (HOW) and involvement of treatment supporters during counselling improved uptake and family-level engagement.

Adherence was strengthened through structured follow-up and community engagement. Early dropout within the first month of treatment (15%–19% during the initial phase of the project) primarily linked to mild adverse drug reaction (ADR) declined with enhanced follow-up, including at least two in-person visits during the first month. Community health workers (ASHAs, Mitanins, Sahiyas) supported adherence through counselling and follow-up across households.

Forecasting tools and continuous monitoring of drug availability reduced stockouts and ensured uninterrupted TPT initiation and continuation.

Long-term follow-up showed <1% progression to active TB post-TPT, with a median time to progression of 10 months (interquartile range: 7–13), and longer median progression among those on 3HP (19 months) compared to 6H (9.5 months), confirming the effectiveness of TPT. However, despite this achievement, TPT uptake in the private sector remained low (17%), indicating persistent challenges in private sector engagement and continuity of preventive care.

## DISCUSSION

The TPT scale-up initiative under the Global Fund GC6 grant made significant progress in identifying and protecting HHCs of PTB patients, while also generating critical technical and operational insights. Substantial TPT uptake and high overall treatment completion, particularly with shorter regimens, reflects the feasibility of integrating preventive services within routine TB care and supports ongoing policy shift toward simplified preventive therapy schedules.

Comparable IGRA positivity among contacts of bacteriologically confirmed and clinically diagnosed TB cases in the JEET 2.0 pilot districts, supported by findings from the Maharashtra sub-analysis (Axshya Plus districts), indicates that TBI is not restricted to bacteriologically confirmed index cases. This suggests that reliance on bacteriological status alone for prioritising preventive therapy may be insufficient and highlights the need to refine risk-based eligibility criteria under national TB programmes to ensure broader and more equitable coverage of at-risk populations. The observed heterogeneity in TBI across geographical settings, including higher IGRA positivity in hilly regions, underscores the influence of contextual factors such as background TB burden, population mobility, and access to diagnostic services. As IGRA positivity is shaped by geographical location, demographic characteristics, sub-national TB incidence, and exposure patterns,^14,15^ TB programmes should adopt context-specific diagnostic and implementation strategies aligned with sub-national disease burden, population mobility, and access barriers.

The project highlighted insights in TBI diagnostic options within the ‘Test and Treat’ approach. IGRA testing, with ∼25% positivity, helped reduce potential over-treatment and optimise resource use.^3,7^ However, its wider adoption in India is limited by cost (∼USD 29 per test). TST showed lower positivity (∼10%) and operational limitations related to training and interpretation. The positivity rate of both IGRA and TST in this project is much lower than the 36% prevalence reported in the general population by a representative systematic review and meta-analysis of TBI in India highlighting potential underdiagnosis and missed opportunities to preventive treatment.^16^ The WHO-endorsed newer antigen-based skin tests such as Cy-Tb offer improved performance and operational feasibility in decentralised settings, though cost-effectiveness for national scale-up requires further evaluation.^3,17^

Integration of CI with ACF improved TB detection among HHCs, including a substantial proportion of asymptomatic cases identified through handheld digital CXRs. This underscores the limitation of symptom-based screening alone and reinforces the value of radiological screening in detecting subclinical disease and ensuring exclusion of active TB prior to TPT initiation. The use of portable CXR in community settings further supports early detection in resource-constrained and geographically remote areas. This approach aligns with WHO guidance on integrating CI and ACF to curb TB transmission and disease progression.^3^

CXR proved to be a critical tool for strengthening diagnostic confidence prior to TPT initiation. Its integration into screening algorithms supports both providers and patients in decision-making and has informed revisions in national guidelines. The increasing availability of AI-assisted CXR further offers opportunities to enhance diagnostic accuracy and triaging in resource-limited settings.^18^ Incorporating such sensitive tools such as AI-enabled CXR can enhance diagnostic accuracy, ensuring appropriate assessment of TPT eligibility. Additionally, integrating community-based service delivery and teleconsultation may further reduce access barriers and out-of-pocket expenditures, particularly in remote and underserved areas.^19–21^

On the treatment side, shorter regimen 3HP was associated with higher adherence and lower attrition compared to longer regimens (6H), consistent with global evidence.^22,23^ Nonetheless, ADRs, even when mild, and inadequate counselling were a major contributor for early treatment discontinuation particularly during the first month of treatment. This highlights the importance of proactive ADR management, structured counselling, and household-level engagement.^24^ While frameworks such as WHO’s WHEN-WHAT-HOW approach provide guidance, variability in implementation highlights the need for strengthening provider capacity and standardising counselling practices.^16,25^ Strengthened monitoring systems including real-time electronic recording and reporting across the TPT care cascade can help identify attrition points and enable timely corrective actions.^26,27^

Operationally, integration of TPT into the primary health care system was a key enabler of programme performance. Leveraging HWCs and community health officers (CHO), along with community health workers such as ASHAs, Mitanins, and Sahiyas, improved access, adherence, and continuity of care. Financial incentives tied to treatment completion further boosted uptake, reinforcing the effectiveness of community-based strategies.^28–31^ However, private sector engagement remained limited, with only 17% of eligible HHCs receiving TPT, highlighting ongoing challenges in public–private integration. Evidence from more successful models suggests that dedicated provider engagement, performance-based incentives, and simplified reporting tools are critical to strengthening private sector participation and expanding coverage.^32,33^

Finally, long-term follow-up findings reinforce the protective effect of TPT when implemented effectively with very low progression to active TB.^34^ These findings highlight the importance of sustaining follow-up beyond treatment completion. Despite national guideline recommending post-TPT follow-up, systematic tracking remains inconsistently implemented, representing missed opportunities for early detection and intervention. Integrating digital tools, such as electronic case tracking platforms and interoperable health information systems, offers a promising solution to strengthen monitoring, enable timely interventions, and improve accountability within TPT programmes.^35^ Scaling up such innovations should be prioritised to sustain the gains achieved through preventive therapy and support broader TB elimination efforts.

## CONCLUSION

This study demonstrates the feasibility and impact of large-scale TPT implementation in high-burden settings when supported by robust health systems and adaptive strategies. Key enablers included structured counselling, decentralised community-based delivery, appropriate diagnostic use, shorter regimens such as 3HP, and integration with ACF to prevent TB and detect undiagnosed cases. Programme challenges, including diagnostic variability, early dropouts due to ADRs, drug stockouts, and limited private sector engagement, underscore the need for sustained programmatic focus. Insights from this project’s initiative have directly informed national scale-up of Cy-TB testing, 3HP expansion, and community health worker incentivisation. Future scale-up efforts must prioritise workforce capacity, counselling, pharmacovigilance, digital tracking, and community engagement. Leveraging innovations such as AI-supported CXR and shorter TPT regimens can further optimise TPT delivery, contributing to India’s TB elimination goals and the broader End TB strategy.
